# Pandemic Acceptance and Commitment to Empowerment Response (PACER) Training: Protocol for the Development and Rapid-Response Deployment

**DOI:** 10.2196/33495

**Published:** 2021-12-06

**Authors:** Kenneth Fung, Jenny JW Liu, Mandana Vahabi, Alan Tai-Wai Li, Mateusz Zurowski, Josephine Pui-Hing Wong

**Affiliations:** 1 Department of Psychiatry Faculty of Medicine University of Toronto Toronto, ON Canada; 2 Toronto Western Hospital University Health Network Toronto, ON Canada; 3 Daphne Cockwell School of Nursing Ryerson University Toronto, ON Canada; 4 Regent Park Community Health Centre Toronto, ON Canada

**Keywords:** COVID, COVID-19, coronavirus, pandemic, resilience, acceptance commitment therapy, group empowerment

## Abstract

**Background:**

During a global pandemic, it is critical to rapidly deploy a psychological intervention to support the mental health and resilience of highly affected individuals and communities.

**Objective:**

This is the rationale behind the development and implementation of the Pandemic Acceptance and Commitment to Empowerment Response (PACER) Training, an online, blended, skills building intervention to increase the resilience and well-being of participants while promoting their individual and collective empowerment and capacity building.

**Methods:**

Based on acceptance and commitment therapy (ACT) and social justice–based group empowerment psychoeducation (GEP), we developed the Acceptance and Commitment to Empowerment (ACE) model to enhance psychological resilience and collective empowerment. The PACER program consists of 6 online, interactive, self-guided modules complemented by 6 weekly, 90-minute, videoconference, facilitator-led, group sessions.

**Results:**

As of August 2021, a total of 325 participants had enrolled in the PACER program. Participants include frontline health care providers and Chinese-Canadian community members.

**Conclusions:**

The PACER program is an innovative intervention program with the potential for increasing resilience and empowerment while reducing mental distress during the pandemic.

**International Registered Report Identifier (IRRID):**

DERR1-10.2196/33495

## Introduction

### Background

The COVID-19 pandemic has had a devastating impact globally, precipitating a mental health crisis. Early data from a global survey in April 2020 to May 2020 found that 58.6% of respondents exhibited some symptoms of depression [1]. Based on a population-based survey from July 2020, it is estimated that over 50% of Canadians reported worsened mental health, while over 81% of workers surveyed reported significant declines in their mental well-being since the onset of the pandemic [2]. While data on suicide rates is not yet clear, predictive models vary widely, anticipating a 1%-145% increase. The reported data on mental health are dynamically changing across countries, calling for proactive steps to promote mental health and prevent suicide, especially among the vulnerable [3].

One of the challenges to addressing this mental health crisis is the complexity of the issues. In addition to psychological anxiety and fear brought on by the pandemic itself, the introduced public health measures, such as social distancing and lockdowns, have had a cascade of deleterious psychosocial and economic sequalae on individuals, families, and communities, ranging from social isolation to housing and financial crisis to disruption of access to community supports and services. These measures have a negative impact on social determinants of health and mental health, amplifying existing inequities [4], further exacerbated by COVID-19–related racism, and resulting in poorer mental health [5]. The possible ramifications and fallout of these factors for mental health will continue to unfold for months and years to come.

### The Acceptance and Commitment to Empowerment (ACE) Model

To promote resilience and address the multidimensional stressors on mental health, we adapted a resilience building model, the Acceptance and Commitment to Empowerment (ACE) model, for pandemic response. ACE integrates the acceptance and commitment therapy (ACT) [6], an evidence and mindfulness-based intervention, with a social justice–based group empowerment psychoeducation (GEP) [7]. While self-help resources and psychological interventions such as positive reframing, rational thinking, and various coping strategies can be helpful, they may add to a sense of futility in a pandemic when many are faced with external stressors and systemic inequities beyond their control. ACT aims to increase psychological flexibility and adaptability by helping individuals to accept their negative thoughts and feelings rather than challenging them or being entangled by them, get in contact with the here and now as individuals not encumbered by labels and judgments, and find meaning and values through persistent efforts and actions. GEP includes 4 explicit core principles: social justice and equity; empathy and compassion; interdependence; and collective empowerment, fostered through critical reflection, critical dialogue, collaborative learning, and experiential learning. GEP promotes a deeper appreciation of our interdependence in the context of mutual empathy, social justice, and health equity. It empowers individuals to build and tap into their social networks and expand their capacity to engage in mutual support and social activism. Combined, the ACE model has been successfully used to decrease stigma and promote mental well-being in various marginalized populations [7] and adapted for pandemic challenges.

### Research Aims

This paper describes the protocol for the development and implementation of the Pandemic Acceptance and Commitment to Empowerment Response (PACER) program. The PACER program was developed during the period of April 2020 to June 2020 and launched in July 2020. As of August 2021, the program was still ongoing, with rolling recruitment and enrollment in cohorts.

## Methods

### Development of the PACER Program

Constrained by lockdowns, we developed the PACER program to be delivered as an online intervention. The PACER program uses the ACE model as the basis of content development. The program consists of 6, 1-hour, weekly, online, self-guided learning modules accompanied by weekly, 90-minute, facilitated, online group videoconferencing sessions. The online modules are hosted on Moodle, an online e-learning platform with open-sourced coding of activities and exercises. Activities within the PACER modules are designed with the help of an external information technology company in consultation with the research team.

**Figure 1:**
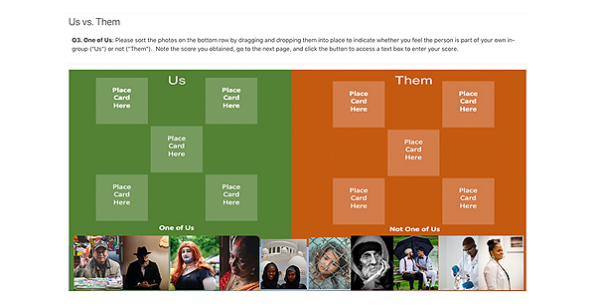
Examples of an interactive Pandemic Acceptance and Commitment to Empowerment Response (PACER) exercise.

The PACER program modules include a variety of interactive activities, such as reflective writing, guided meditation, and gamified exercises to explore ACE principles and processes. As an example, while most of our gamified exercises do not include a scoring scheme, in one particular acceptance exercise, participants are repeatedly presented with a stressful scenario and asked to select a response from a variety of coping strategies, such as having balanced thinking, doing physical and relaxation exercises, and practicing mindfulness meditation to earn points. Each time any coping strategy is chosen, points are awarded, but this promptly returns the participant back to the starting point of the scenario. The points are actually arbitrary and meaningless, serving only as a behavioral reinforcer to have participants persist in this loop, which continues until the participant finally gives up on finding “the right solution” and chooses the exit option. The debriefing text then guides reflection on a potential trap that we may fall into when we try to avoid and eliminate negative emotions, as many coping strategies may seem rewarding in the short term. In this regard, even mindfulness practice may function as an avoidant behavior. In another exercise, the participant is prompted to categorize people into an in-group versus an out-group to confront their own unconscious process of “othering” and discuss its role in society, including inequities during the pandemic (see Figure 1).

These activities facilitate learning by integrating 3 aspects: the actual present moment experience of the participants’ engagement and interaction online; reflection on personal and communities’ real-life struggles with the pandemic and its sequalae; and the application of ACE theory, principles, and skills. Each module begins with the personal and builds towards a broader collective and systemic perspective. The journey helps participants to connect more deeply with their values and purpose, with others in interpersonal relationships, and with the community at large. The modules increase in complexity across the 6 modules with the final sets of exercises tapping into multiple principles and skills, culminating in developing effective personal and community-level actions to promote individual and collective mental well-being.

The weekly online group videoconferencing is led by trained facilitators and provides an opportunity for sharing, collective reflection, and group discussion of the weekly learning activities. It reinforces and broadens the learning, as each participant may have a different interpretation of and insights into the activities, informed by their own pandemic struggles. It also fosters mutual support in dealing with personal and societal pandemic stressors.

### Participant Selection and Recruitment

The PACER training program prioritizes 2 populations: frontline health care providers (HCPs) and members of the Chinese-Canadian communities (CH-COM). Frontline HCPs are at high risk of burnout related to increased workload, fear of contracting the illness and spreading it to their family, shifting roles and work protocols, and compassion fatigue in caring for the sick and dying. Members of CH-COM have been experiencing intensified social marginalization, racial discrimination, and pandemic-related stigma in addition to pandemic stress. A Canadian national survey showed that 64% of Chinese-Canadians had experienced some form of racism since the onset of the pandemic [8]. To accommodate differences in lived experiences of both groups, we developed 2 arms of the program. While the content and exercises remain the same for both arms, instructions and examples throughout are adjusted to be more relevant for the intended audiences.

The recruitment of PACER participants is conducted through convenience and snowball sampling. Recruitment methods include outreach to hospital and health care networks and Chinese community agencies using promotional e-flyers. In addition to targeted promotion, recruitment also involved word of mouth, social media (Facebook, Instagram, Twitter, WeChat), and promotional videos posted on YouTube. Eligible participants include anyone identifying as a health care worker and/or member of Asian Canadian communities. Participant enrollment in active cohorts is prioritized based on level of distress and availability to attend the predetermined weekly facilitated discussions. Participants low in distress or are not available for the weekly discussions are put on a waitlist for future cohorts. No eligible participant will be deterred from participation.

### Procedure

Eligible participants are invited to indicate their language preferences (English, Chinese Mandarin, Chinese Cantonese), current level of distress, and availabilities to attend weekly discussions in the evenings or on weekends. Following, participants are matched with a cohort led by 2 experienced facilitators. Once enrolled, participants are invited to complete a set of pre-intervention measures. During each week of the 6-week PACER program, participants also complete weekly responses prior to their weekly facilitated discussions. After the completion of the program, participants are invited to a set of self-report measures immediately postprogram and at 3-months follow-up. Weekly and biweekly activity logs are used to track participants’ activities in mental health promotion from postintervention to the 3-month follow-up. A focus group will be conducted at follow-up to gather their reflections on their experience of the program. The research ethics boards at Ryerson University, York University, and the University Health Network – Toronto Western Hospital approved the study. All study participants provided informed consent prior to participation.

### Measures

To evaluate the effectiveness of the PACER program, participants complete a set of online self-report questionnaires. The questionnaires include demographic and background questions constructed for the purpose of the current study, as well as validated self-report instruments evaluating dimensions of mental health, resilience, and well-being. The measures are described in the following sections.

#### Demographic and Background Information

Participants will complete information on age, gender, education, ethnoracial identity, status in Canada, professional role(s), workplace setting, and preferred language. Questions are generated for the purpose of the current study.

#### General Mental Distress

To measure general mental health and distress, participants will complete the General Health Questionnaire (GHQ) [9]. The GHQ is a 12-item, self-report screening tool for general mental distress and general (nonpsychotic) mental health symptoms.

#### Psychological Flexibility

To measure psychological flexibility, participants will complete the Acceptance and Action Questionnaire II (AAQ-II) [10]. The AAQ-II is a 7-item, self-report measure of psychological inflexibility.

#### Resilience

To measure resilience, participants will complete the Multi-System Model of Resilience Inventory (MSMR-I) [11]. The MSMR-I is a 27-item, self-report measure of resilience capacity across 3 systems of resilience resources (internal, coping and pursuits, and external).

#### Empowerment

To measure empowerment, participants will complete the Empowerment Scale (ES) [12]. The ES is a 33-item, self-report measure of empowerment capacity.

### Data Analysis Plan

Quantitative data will be aggregated, and descriptive and change-based data will be analyzed using SPSS (IBM Corp, Armonk, NY). Data analyses include descriptive statistics, measures of internal consistency, and analysis of variance. Qualitative data (from focus groups) will be transcribed verbatim. N-Vivo software will be used for data management. Data will be organized and analyzed using thematic and focused descriptive analysis using inductive and deductive approaches.

## Results

As of August 2021, 325 participants have been enrolled in cohorts in English, Chinese Mandarin, and Chinese Cantonese. Of those enrolled, 287 have completed the full training, and 38 participants have dropped out due to scheduling conflicts. To date, no adverse events have been reported by participants taking part in the PACER training program. This study is expected to conclude in April 2022.

## Discussion

The COVID-19 pandemic has created great mental health challenges. It is imperative to develop accessible and effective interventions to strengthen mental health resilience of affected populations. A wide spectrum of psychological interventions, from psychodynamic therapy to existential therapy to cognitive behavioral therapies, have been proposed [13]. Here, we outlined the protocols for the development and implementation of an online blended learning intervention that combines ACT with GEP to form the ACE model.

Our ACE model is unique in bringing together an individual psychological intervention with a collectivistic social justice intervention. This integration increases its suitability to address an external mass event like the pandemic, which has disrupted social connections, threatened our way of living, and led to worsening of pre-existing inequities and racism. Based on the ACE model’s mindfulness approach, there is an emphasis on the acceptance of negative thoughts and feelings, rather than focusing on attempts to avoid or reframe them positively, rationally, or realistically. It presents a palatable approach for many health care professionals who have experienced guilt about their own distress and in the role transition from being a helper to receiving help, with their initial mindset that they ought to have been more competent, rational, and resilient. The ACE model’s focus on mindful acceptance also departs from preoccupation with coping, as emphasis on coping strategies may paradoxically add to further stress experienced. In addition, many have expressed frustration and powerlessness in dealing with various health and systemic policies that are a departure from the norm and implemented rapidly in a continuously changing landscape leading to inefficiencies and perceived increased health risks for HCPs and patients, as well as health disparities. Along similar lines, marginalized and discriminated community members report experiences of shame, powerlessness, internalized stigma, and/or a sense of burden to stand up against social injustice. The ACE model encourages a nonjudgmental acceptance stance towards these negative thoughts and feelings, decreasing denial and easing the burden of justification and shame, while identifying the real-world impact of the pandemic, fostering a sense of shared social responsibility, and empowering all for collective action.

Our delivery method is uniquely suited for the pandemic situation. At a time when personal existential issues and larger social justice issues have been elevated to the forefront, the program is designed to foster both individual and collective deep reflection. In addition to being physically safe and pragmatically feasible, the self-guided interactive exercises are innovative in encouraging participants to learn from their own experiences and apply them to issues that are deeply personal, sensitive, or even controversial at a societal level. When these personal experiences are shared and disclosed in a safe space created by the virtual group, participants are empowered and appreciate the powerful mutual learning and support, consistent with the widening of an individual focus to the collective.

We anticipate the data collected will provide important insights regarding the effectiveness of PACER, as well as distinguish the pathways of change in different population groups. The PACER intervention, if found to be effective, may be adapted for other communities and the population at large. While we prioritized frontline HCPs and CH-COM members in this study, we anticipate that PACER can be contextualized for deployment and use with other populations to support their unique and shared challenges. As we complete the study to determine our particular intervention’s effectiveness, we advocate for public health and mental health providers to urgently focus more on effective mental health interventions and initiatives that promote psychological acceptance and flexibility rather than rationalization and advance collective well-being rather than the more limited concept of self-care. With the notion of an independent self being broadened to an interdependent self, the concept of self-care becomes inextricably linked with caring for the communities as a whole. We need to develop a sense of community cohesion while acknowledging our individual differences brought on by differences in power and privilege. This can be likened to not only weathering the same storm together but also being on the same boat that is humanity, even though we need to acknowledge and redress the fact that we inhabit different deck levels.

## Abbreviations

AAQ-II: Acceptance and Action Questionnaire II
ACE: Acceptance and Commitment to Empowerment
ACT: acceptance and commitment therapy
CH-COM: Chinese Canadian community
ES: Empowerment Scale
GEP: group empowerment psychoeducation
GHQ: General Health Questionnaire
HCP: health care professional
MSMR: Multi-System Model of Resilience
PACER: Pandemic Acceptance and Commitment to Empowerment Response
